# Atypical Leber Hereditary Optic Neuropathy (LHON) Associated with a Novel MT-CYB:m.15309T>C(Ile188Thr) Variant

**DOI:** 10.3390/genes16010108

**Published:** 2025-01-20

**Authors:** Sanja Petrovic Pajic, Ana Fakin, Martina Jarc-Vidmar, Maja Sustar Habjan, Lucija Malinar, Kasja Pavlovic, Nina Krako Jakovljevic, Andjelka Isakovic, Sonja Misirlic-Dencic, Marija Volk, Ales Maver, Gregor Jezernik, Damjan Glavac, Borut Peterlin, Ivanka Markovic, Nebojsa Lalic, Marko Hawlina

**Affiliations:** 1 Eye Hospital, University Medical Centre Ljubljana, 1000 Ljubljana, Slovenia; sanja.petrovic-pajic@med.bg.rs (S.P.P.); ana.fakin@gmail.com (A.F.); martina.jarcvidmar@gmail.com (M.J.-V.); sustar.majchi@gmail.com (M.S.H.); laralucija2@gmail.com (L.M.); 2 Clinic for Eye Diseases, University Clinical Centre of Serbia, 11000 Belgrade, Serbia; 3 Faculty of Medicine, University of Belgrade, 11000 Belgrade, Serbia; 4 Faculty of Medicine, University of Ljubljana, 1000 Ljubljana, Slovenia; 5 Clinic for Endocrinology, Diabetes and Metabolic Diseases, University Clinical Center of Serbia, 11000 Belgrade, Serbia; kasja.pavlovic@med.bg.ac.rs (K.P.); ninakrako@gmail.com (N.K.J.); 6 Institute of Medical and Clinical Biochemistry, Faculty of Medicine, University of Belgrade, 11000 Belgrade, Serbia; andjelka.isakovic@med.bg.ac.rs (A.I.); sonja.misirlic-dencic@med.bg.ac.rs (S.M.-D.); ivanka.markovic@med.bg.ac.rs (I.M.); 7 Center of Excellence for Redox Medicine, 11000 Belgrade, Serbia; 8 Clinical Institute of Genomic Medicine, University Medical Centre Ljubljana, 1000 Ljubljana, Slovenia; marija.volk@kclj.si (M.V.); ales.maver@kclj.si (A.M.); borut.peterlin@kclj.si (B.P.); 9 Center for Human Genetics and Pharmacogenomics, Faculty of Medicine, University of Maribor, 2000 Maribor, Slovenia; gregor.jezernik1@um.si (G.J.); damjan.glavac@mf.uni-lj.si (D.G.); 10 Department of Molecular Genetics, Faculty of Medicine, University of Ljubljana, 1000 Ljubljana, Slovenia; 11 Department of Medical Sciences, Serbian Academy of Sciences and Arts, 11000 Belgrade, Serbia; lalic.nm@gmail.com

**Keywords:** LHON, *MT-CYB*:c.563T>C p.(Ile188Thr), chrM:15309T>C, *MT-CYB* gene, electrophysiology, retinal segmentation, VA improvement, mitochondrial disfunction, proteomic analysis

## Abstract

**Background:** The study presents a detailed examination and follow-up of a Slovenian patient with an Leber Hereditary Optic Neuropathy (LHON)-like phenotype and bilateral optic neuropathy in whom genetic analysis identified a novel variant *MT-CYB*:m.15309T>C (Ile188Thr). **Methods:** We provide detailed analysis of the clinical examinations of a male patient with bilateral optic neuropathy from the acute stage to 8 years of follow-up. Complete ophthalmological exam, electrophysiology and optical coherence tomography (OCT) segmentation were performed. The genotype analysis was performed with a complete screening of the mitochondrial genome. Furthermore, proteomic analysis of the protein structure and function was performed to assess the pathogenicity of a novel variant of unknown significance. Mitochondrial function analysis of the patient’s peripheral blood mononuclear cells (PBMCs) was performed with the objective of evaluating the mutation effect on mitochondrial function using flow cytometry and high-resolution respirometry. **Results:** The patient had a profound consecutive bilateral visual loss at 19 years of age due to optic neuropathy with characteristics of LHON; however, unlike patients with typical LHON, the patient experienced a fluctuation in visual function and significant late recovery. He had a total of three visual acuity deteriorations and improvements in the left eye, with concomitant visual loss in the right eye and a final visual acuity drop reaching nadir 9 months after onset. The visual loss was characterized by centrocecal scotoma, abnormal color vision and abnormal VEP, while deterioration of PERG N95 followed with a lag of several months. The OCT examination showed retinal nerve fiber layer thinning matching disease progression. Following a two-year period of legal blindness, the patient’s visual function started to improve, and over the course of 5 years, it reached 0.5 and 0.7 Snellen (0.3 and 0.15 LogMAR) visual acuity (VA). Mitochondrial sequencing identified a presumably pathogenic variant m.15309T>C in the *MT-CYB* gene at 65% heteroplasmy, belonging to haplogroup K. Mitochondrial function assessment of the patient’s PBMCs showed a lower respiration rate, an increase in reactive oxygen species production and the presence of mitochondrial depolarization, compared to an age- and sex-matched healthy control’s PBMCs. **Conclusions:** A novel variant in the *MT-CYB*:m.15309T>C (Ile188Thr) gene was identified in a patient with optic nerve damage and the LHON phenotype without any additional systemic features and atypical presentation of the disease with late onset of visual function recovery. The pathogenicity of the variant is supported by proteomic analysis and the mitochondrial dysfunction observed in the patient’s PBMCs.

## 1. Introduction

Leber Hereditary Optic Neuropathy (LHON) mostly occurs as a result of one of the three-point mutations in mtDNA located at nucleotides 11,778, 14,484 and 3460, all affecting the NADH dehydrogenase (ND) subunits of complex I of the electron transport system (ETS) [1,2]. Primary mutations are mostly homoplasmic (100% of mtDNA is mutated), but there are some instances of heteroplasmic cases also [3,4].

Some 3–11% of LHON or LHON-plus phenotypes are linked to several other mutations [1,2,5]. Most of these are typically found in the ND1, ND4 and ND6 genes, which are considered mutational “hotspots” [6]. These mutations are regarded as rare mutations [7], and they are considered pathogenic if impaired protein and mitochondrial function can be confirmed by proteomic and mitochondrial function analysis. At present, some 125 different point mutations have been associated with LHON. A total of 18 of these mutations have been confirmed as pathogenic for LHON (https://www.mitomap.org/MITOMAP, accessed on 18 November 2025). Such mutations usually occur within individual families or individuals and are mostly heteroplasmic. However, genetically unconfirmed cases with a typical LHON phenotype are a diagnostic challenge [8].

Pathogenic variants (nonsense, missense or frameshift) in the *MT-CYB* gene leading to the complex III deficiency have been reported in severe exercise intolerance, myopathy, encephalopathy, cardiomyopathy, septo-optic dysplasia and multisystem disorders [9]. There are 72 mutations reported in *MT-CYB*, out of which 20 have been reported in LHON (14), MELAS (4) and Leigh syndrome (2) (https://www.mitomap.org/MITOMAP, accessed on 18 November 2025).

Herein we present a novel m.15309T>C (Ile188Thr) variant in the *MT-CYB* gene associated with isolated optic nerve damage (LHON phenotype) without extraocular features. Additionally, disease course was atypical with fluctuating vision in the early stages and late onset of visual recovery (2 years). As assessed by proteomic analysis and mitochondrial function studies of the LHON patient’s peripheral blood mononuclear cells (PBMCs) and the PBMCs of a healthy age- and sex-matched control, we show that this variant might be the cause of the patient’s mitochondrial dysfunction.

## 2. Materials and Methods

Patients provided written informed consent according to regulations of University Medical Centre Ljubljana; the use of clinical data was approved by the National Committee for Medical Ethics of Slovenia (No: 0120-626/2019/5, date: 17 March 2020) and the Committee for Medical Ethics, Medical Faculty, University of Belgrade, Serbia (No: 1322/V-11, date: 28 May 2020). All patients received all examinations as routine diagnostic work-up. Written consent for whole-blood collection of the patient and an age- and sex-matched control was also provided.

### 2.1. Ocular Examination

**Figure 1:**
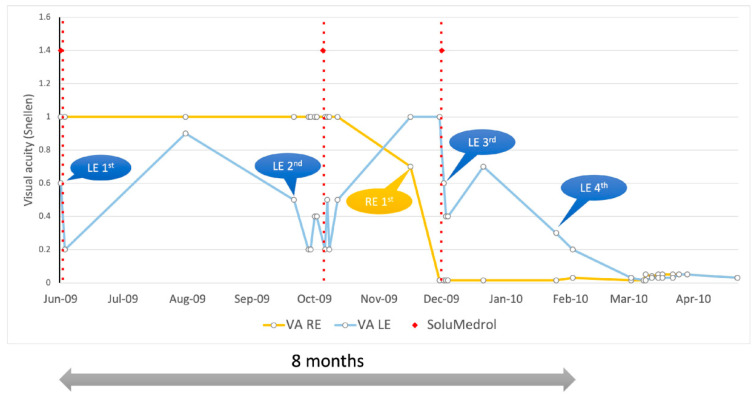
Visual acuity fluctuations during first eight months since disease onset.

A patient with LHON-like optic neuropathy together with his three unaffected first-degree relatives (two sisters and mother) are presented. The family pedigree is shown in Figure 1. The affected patient experienced bilateral sequential visual acuity loss. He had a detailed diagnostic work-up to exclude other optic neuropathies: glaucoma, compressive, demyelinating, inflammatory, infective, infiltrative or toxic causes. The head MRI scan was normal. Ophthalmological examinations were performed at the presentation as well as during follow-up periods and included best-corrected visual acuity (Snellen), color vision (Ishihara plates), visual field examination (Goldmann or Octopus perimetry), fluorescein angiography (FA) and electrophysiology testing. Ring analysis of the retinal nerve fiber layer around the optic disk with spectral domain optical coherence tomography (SD-OCT) and segmentation of the retinal layers were also carried out at presentation and follow-up visits. SD-OCT segmentation was performed by the Spectralis HRA apparatus (Heidelberg Engineering, Heidelberg, Germany) according to the protocol described previously [10,11]. The software automatically provided an Early Treatment Diabetic Retinopathy Study (ETDRS) retinal thickness map of the layers in accordance with the consensus of the International Nomenclature for OCT. Inadequately defined layers were manually revised. The results were compared with the published standards using the same method [10]. Electrophysiological testing with large-field pattern electroretinogram (PERG) and visual evoked potentials (VEPs) were recorded with an Espion visual electrophysiology testing system (Diagnosys LLC, Littleton, MA, USA), while full-field ERGs were recorded using the RetiPort system (Roland Consult, Brandenburg, Germany). All electrophysiological tests followed the standard of the International Society for Clinical Electrophysiology of Vision (ISCEV) [12,13,14].

### 2.2. Genetic Analysis

Genetic analysis was performed with a next-generation sequencing approach on an Ion Torrent PGM (Life Technologies, Carlsbad, CA, USA) on whole mtDNA. Torrent Suite 2.2 software was used to map and align sequences for barcoded samples to the mtDNA reference sequence (rCRS -NC_012920).

We first identified the loci of the identified variants to exclude the variations in the non-coding regions. The coding effect was determined using MitoTool software, V 1.1.2. For the final computational analysis, we used only non-synonymous variations found in the LHON samples.

In addition, whole-exome sequencing was performed in all family members, focusing on known genes associated with optic neuropathy (Panel Optic neuropathy v3.0, Panelapp, Genomics England).

### 2.3. Mitochondrial Function Assessment

To confirm the pathogenicity of the confirmed genetic variant, we assessed mitochondrial function of peripheral blood mononuclear cells (PBMCs) from this patient and a sex- and age-matched control. We also conducted analysis of the protein structure and function relationship.

PBMCs from the patient and a sex- and age-matched control (C1) were collected from 4 × 6 mL of heparinized whole-blood samples. Whole anticoagulated blood was centrifuged, and the superficial part of the cell pellet from each vacutainer was collected and processed using Ficoll density gradient centrifugation. PBMCs were collected, washed and counted.

PBMCs’ mitochondrial function was assessed using high-resolution respirometry (Oroboros Oxygraph-2k, Oroboros Instruments, Innsbruck, Austria, DatLab 4 software). After isolation and a freezing–thawing procedure, PBMCs were resuspended in mitochondrial respiration medium MiR05 (EGTA 0.5 mM, MgCl_2_ × 6H_2_O 3 mM, lactobionic acid 60 mM, taurine 20 mM, KH_2_PO_4_ 10 mM, HEPES 20 mM, D-sucrose 110 mM, BSA 1 g/L, pH 7.1), and all the measurements in the O2k were carried out in the same medium. The cell number per chamber and digitonin concentration (5–7 × 10^6^ cells/chamber; 8 µg digitonin/10^6^ cells) were optimized prior to the experiments according to standard protocols. Substrate uncoupler inhibitor titration (SUIT) protocol was performed and cell respiration was expressed as O_2_ flow per 10^6^ cells [pmol/(s × 10^6^ cells)] in intact cells (ROUTINE) after permeabilization with digitonin and the addition of pyruvate (5 mM), malate (2 mM) and glutamate (10 mM) (LEAK), ADP (2.5 mM) and MgCl (1.5 mM) (oxidative phosphorylation—OXPHOSCI), succinate (10 mM) (OXPHOSCI+CII), rotenone (0.5 µM) (OXPHOSCII) or antimycin A (2.5 µM) (residual oxygen consumption—ROX). All respiratory rates were corrected for ROX. All reagents for high-resolution respirometry were purchased from Sigma-Aldrich (Taufkirchen, Germany).

For flow cytometry analysis, PBMCs were seeded in 24-well plates (2–4 × 10^5^ cells/well) and rested for 1 h to recover. Following appropriate staining, according to manufacturers’ protocols, flow cytometry analysis was performed using an FACSCalibur flow cytometer (BD Biosciences, Heidelberg, Germany) and CellQuest Pro software v.3.3 for acquisition and analysis. The intracellular levels of ROS were measured by flow cytometric analysis of cells stained with dihydrorhodamin 123 (DHR) and dihydroethidium (DHE), a fluorochrome moderately selective for superoxide anions (both from Thermo Fisher Scientific, Waltham, MA, USA). The overall ROS and superoxide ion production was quantified by measuring the intensity of green (FL1) or red (FL2) fluorescence emitted by intracellular ROS or superoxide, respectively, and the results are presented as the fold change in mean fluorescence intensity in comparison to control cells. Mitochondrial membrane potential was assessed using JC-1 (R&D Systems, Minneapolis, MN, USA), a lipophilic cation that forms orange/red fluorescent aggregates upon binding to polarized mitochondria. If the potential is disturbed, the dye cannot access the transmembrane space and remains or reverts to its green monomeric form. The PBMCs were stained with JC-1 as described by the manufacturer, and the green monomer and the orange/red aggregates were detected by flow cytometry.

### 2.4. Protein Structure and Function Analysis

The mitochondrial cytochrome b (MT-CYB) protein sequence, with primary accession P00156, was retrieved from the UniProt database and used to generate AlphaFold2 (AF2) models for both the wild type and the p.I188T variant. The structural effects of the variant were analyzed using PyMOL visualization software V 2.6 by aligning the AF2-generated pathogenic variant MT-CYB models to the crystallographic structure of complex III (PDB: 5XTE) to assess changes in the protein transmembrane region. Hydrophobicity analysis was performed using Kyte–Doolittle hydrophobicity values, derived with ProtScale and plotted using Python’s Matplotlib library to visualize alterations in hydrophobicity at the mutation site. In addition, the impact of the same amino acid substitution using another tool, PROVEAN software V 1.1.3, was predicted.

## 3. Results

### 3.1. Ocular Features

**Figure 2:**
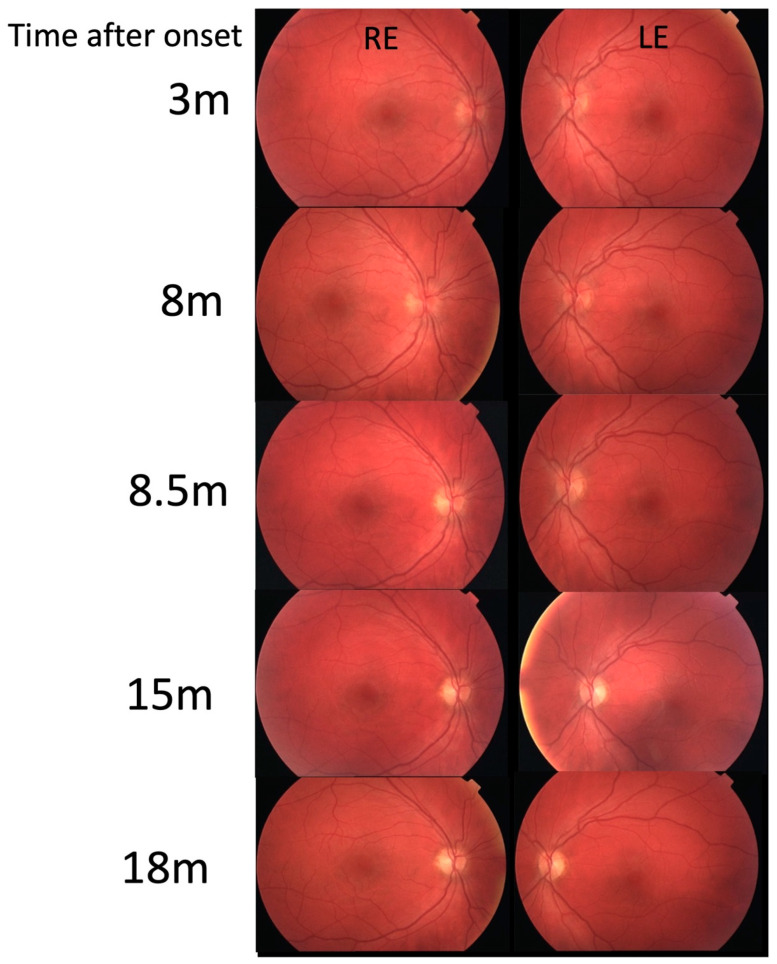
Fundoscopy appearance of our patient. Note the hyperemic optic disks during first eight months since disease onset: only later did the optic disk pallor appear.

We present the case of a 19-year-old male, otherwise healthy, with no family history of ocular disease. In June 2009, the patient noticed poor vision in the left eye and was referred to a local hospital. At that point, the left eye (LE)’s visual acuity (VA) was 0.6 Snellen (0.22 LogMAR) (Figure 1). He was treated for three days with methylprednisolone sodium succinate i.v. (1 g/day) and his VA in the LE worsened further to 0.2 (0.7 LogMAR) (Figure 2). He received antibiotic therapy for suspected sinusitis. In the course of one month, LE VA improved to 0.9 Snellen (0.05 LogMAR). In July 2009 (1 month after the onset), he was seen at the University Eye Hospital Ljubljana, and his VA in the right eye (RE) was 1.0 Snellen (0 LogMAR) and LE was 0.9 Snellen (0.05 LogMAR), while his color vision Ishihara in the RE was 15/15 and LE was 9/15. At this time, MRI of the head and cervical spine was normal.

In September 2009 (2 months later), LE vision deteriorated again (Figure 1). LE VA fluctuated daily from 0.2 to 0.4 Snellen (0.7–0.4 LogMAR) and color vision was 3–9/11 Ishihara. Fundoscopy showed hyperemic optic disks (Figure 2).

**Figure 3:**
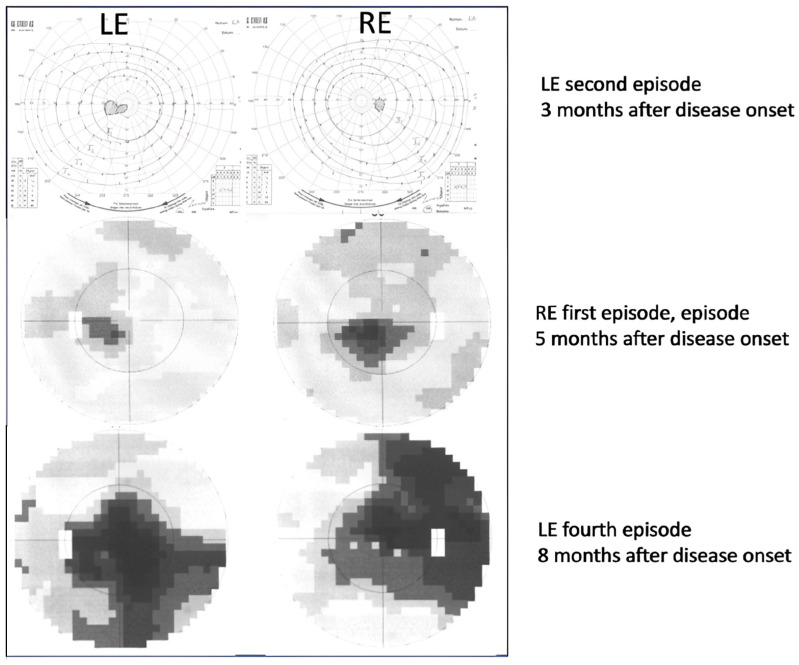
Visual field scotomas with the disease progression (Goldmann—uppermost picture; Octopus G2TOP program—lower two pictures).

There was also small scotoma in the visual field (Figure 3).

Serology excluded possible infectious causes, although he had positive IgM for CMV, which was negative upon repeated testing. He was again treated with methylprednisolone sodium succinate i.v. (1 g/day) for 3 days with no improvement. He also received a prophylactic dose of valacyclovir 250 mg t.i.d. for two weeks due to peroral herpes. Gradually, his LE VA improved to 0.5 Snellen (0.3 LogMAR) with an Ishihara of 11/11. Electrophysiology showed normal PERG and reduced and delayed VEP in the left eye. Two months later (November), the left eye function returned to normal (VA 1.0 Snellen (0 LogMAR); Ishihara 15/15). Soon after that, the patient noticed RE visual decline which in two weeks deteriorated to counting fingers (CF) or 0.0025 Snellen (2.6 LogMAR) (Figure 1) with an Ishihara of 0/15. His optic disks were still pink (Figure 2) and he had central scotoma in the visual field (Figure 3). He again received methylprednisolone sodium succinate i.v. (1 g/day) for 3 days and prophylactic valacyclovir (Valtrex), but there was no improvement in the RE. Surprisingly, upon initiation of corticosteroid therapy, he again experienced deterioration in the previously improved LE to a VA of 0.4 Snellen (0.4 LogMAR), while Ishihara was still 11/11. Electrophysiology showed normal PERG, while VEP was reduced and delayed in both the RE and LE. Three weeks after treatment (end of December 2009), VA in the RE was CF (0.0025 Snellen; 2.6 LogMAR) and LE was 0.7 Snellen (0.15 LogMAR) (slight improvement) with an Ishihara in the RE of 0/15 and in the LE of 11/15. MRI showed no signs of demyelination (Supplementary Figure S1). There were some hyperintense signals along the optic nerves R > L and small effusion within the ON sheath consistent with possible perineuritis of both optic nerves R > L. Lumbar puncture results were normal. One month later, vision in the left eye started to decline again. In two months (8 months since onset), it declined to VA: RLE CF (0.0025 Snellen; 2.6 LogMAR); Ishihara: RE 1/11, LE 2/11 (Figure 1). He also received intravenous immunoglobulins (Octagam) with no improvement. Optic disks were still pink/hyperemic (Figure 2), with large central scotomas in both visual fields (Figure 3). Electrophysiology (10 months after onset) showed a reduction in the PERG N95; VEP amplitude and peak time were severely abnormal. Color vision deteriorated to 1–2/11 in both eyes. The patient reached his nadir visual acuity of CF (0.0025 Snellen; 2.6 LogMAR) in 8 months; optic disks were pink/hyperemic the whole time (Figure 3). Only later did disk pallor begin to appear.

#### Follow-Up Results

**Figure 4:**
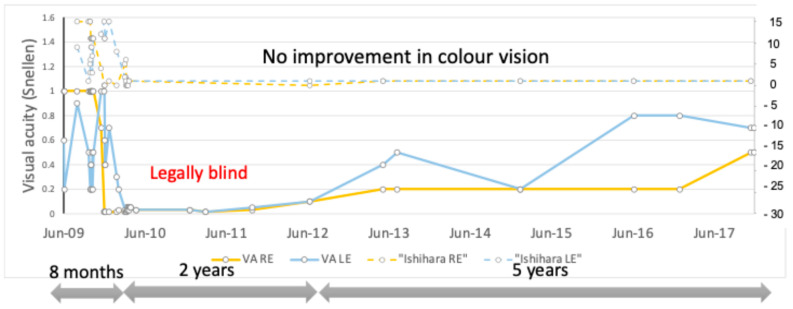
Visual acuity and color vision changes during the follow-up period.

The patient remained legally blind for two years. After that, visual acuity started to improve. Over the course of 5 years, VA reached 0.5 Snellen in the RE and 0.7 Snellen in the LE (0.3 and 0.15 LogMAR) (Figure 4). There was no improvement in color vision during the whole follow-up period of 8 years.

**Figure 5:**
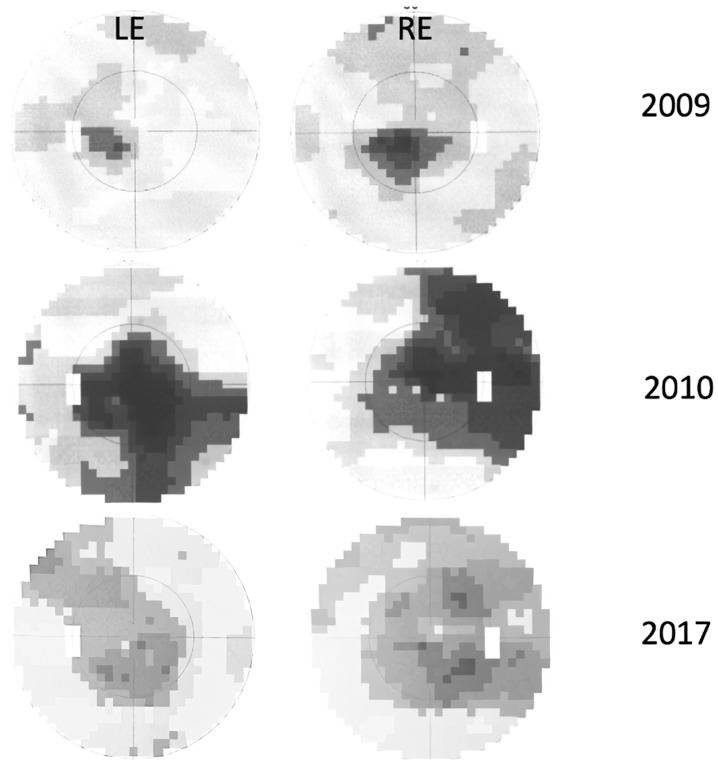
Visual field changes over time (Octopus G2TOP) showing less dense centrocecal scotomas with islands of normal sensitivity and improvement of visual acuity.

Visual fields improved as well, with a reduction in the depth of suppression of the central scotoma (Figure 5).

Microperimetry (Nidek MP1, fast strategy) showed improved fixation, and more stimuli were detected (Supplementary Figure S2). We were interested in identifying whether there was an improvement in GCC thickness in the areas that showed improvement in sensitivity on the microperimetry. Optical coherence tomography showed no structural improvement, not even in the places where there was improvement in the microperimetry (Supplementary Figures S3 and S4). RNFL thickness around optic disks remained low, but there was a bilaterally relatively preserved inferior nasal sector (Supplementary Figure S5).

**Figure 6:**
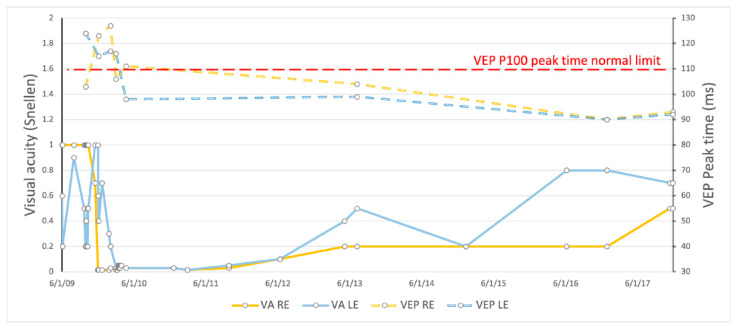
Shortening of the VEP peak time with visual acuity improvement.

Electrophysiology showed a shortening and gradual normalization of the VEP peak time (Figure 6) but no significant improvement in VEP amplitude (Supplementary Figure S6).

PERG N95 was abnormal throughout the follow-up period of eight years. PERG N95 and PERG P50 amplitude showed slight gradual amplitude increase with visual improvement (also probably due to better fixation) (Supplementary Figures S7 and S8).

### 3.2. Genetic Testing Results

Whole-exome sequencing was carried out in all family members and revealed no known pathogenic or novel variants in genes associated with optic neuropathy (Panel Optic Neuropathy, panelapp.genomicsengland.co.uk).

Mitochondrial sequencing showed a novel *MT-CYB*:m.15309T>C (Ile188Thr) missense variant in 65% heteroplasmy in the patient’s peripheral blood mononuclear cells. The same mitochondrial variant, but at different levels of heteroplasmy, has been confirmed in the patient’s mother and both sisters: 38%, 47% and 23%, respectively (I-2, II-1, II-3; Supplementary Figure S9). The identified variant has been previously submitted in the ClinVar database as a variant of unknown significance in a patient with unknown affected status (ClinVar Variation ID: 693862). In silico prediction tools gave conflicting results for this variant (Apogee2—variant of unknown significance; Polyphen—probably damaging; CADD—4.086; GERP 2.04—moderately conserved). The currently available evidence is insufficient to determine the clinical significance of the identified variant. To further elucidate the putative impact of identified variant m.15309T>C in the *MT-CYB* gene, we performed structural modeling and in vitro mitochondrial function testing.

### 3.3. Mitochondrial Function Testing Results

PBMCs from the LHON patient and an age- and sex-matched healthy control were isolated, and mitochondrial function was assessed using high-resolution respirometry and flow cytometry after specific staining. The obtained results indicate that the LHON patient’s PBMCs have lower respiration, produce more reactive oxygen species and show mitochondrial membrane depolarization.

**Figure 7:**
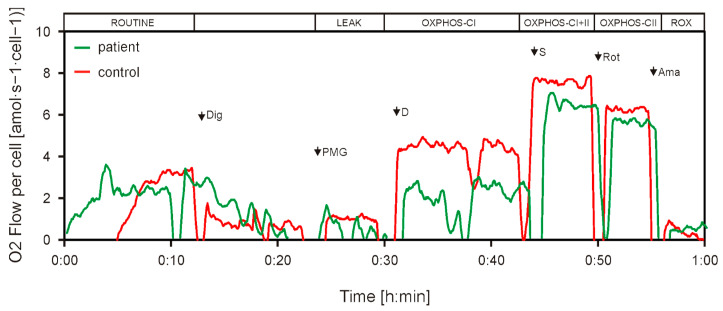
Mitochondrial respiration in the LHON patient’s PBMCs is lower compared to a healthy control. Representative overlapped traces are presented, with different respiratory states: ROUTINE, LEAK, OXPHOS CI, OXPHOS CI+II, OXPHOS CII and ROX. Dig—digitonin; P—pyruvate; M—malate; G—glutamate; D—ADP; S—succinate; Rot—rotenone; Ama—antimycin A.

Respirometry analysis shows lower respiration of the patient’s PBMCs compared to the healthy control’s PMBCs in the ROUTINE and OXPHOS CI respiratory states, which represent respiration of intact cells based on endogenous substrates and oxidative phosphorylation capacity with complex I-linked substrates (Figure 7). Respiration of the patient’s PBMCs was slightly lower compared to the control in the OXPHOS CI+II state, which is consistent with a lower OXPHOS CI capacity, while no difference was observed in phosphorylation capacity with complex II-linked substrates (OXPHOS CII respiratory state) between the patient and control PBMCs.

**Figure 8:**
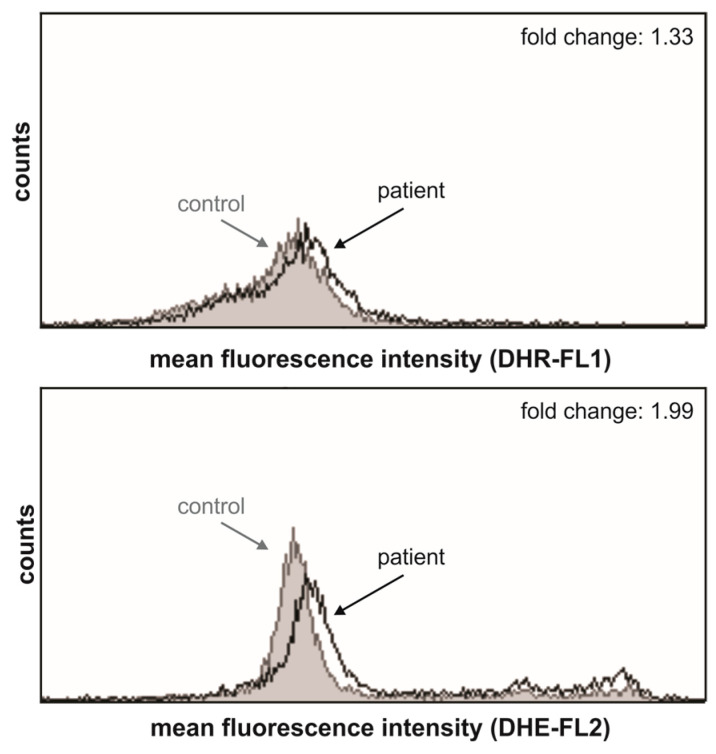
The LHON patient’s PBMCs produce more reactive oxygen species compared to those of the healthy control. Representative histograms from two independent experiments are shown. The ROS and superoxide production, determined by flow cytometry after DHR and DHE staining, respectively, was higher in the PBMCs collected from the patient with LHON compared to the healthy control in which the production was arbitrarily set to 1.

Furthermore, the LHON patient’s PBMCs were in oxidative stress, evidenced by the increased production of ROS and superoxide ions especially (Figure 8), compared to the healthy control’s PBMCs.

**Figure 9:**
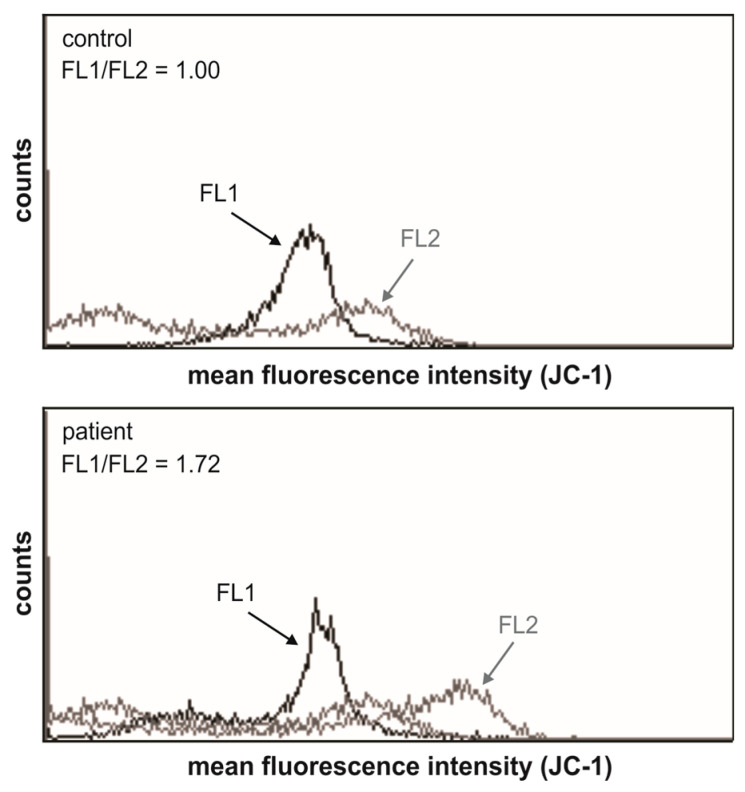
Mitochondrial membrane of LHON patient’s PBMCs is depolarized compared to the healthy control. Representative histograms from two independent experiments are shown. Mitochondrial membrane potential (ΔΨm) of healthy control’s PBMCs was arbitrary set to 1. LHON patient’s PBMCs show an increase in mean fluorescence FL1/FL2 ratio, indicating depolarization.

The LHON patient’s PBMCs also showed depolarization of the mitochondrial membrane, evidenced as an increase in FL1/FL2 mean fluorescence intensity compared to the healthy control’s PBMCs (Figure 9).

### 3.4. Protein Analysis

**Figure 10:**
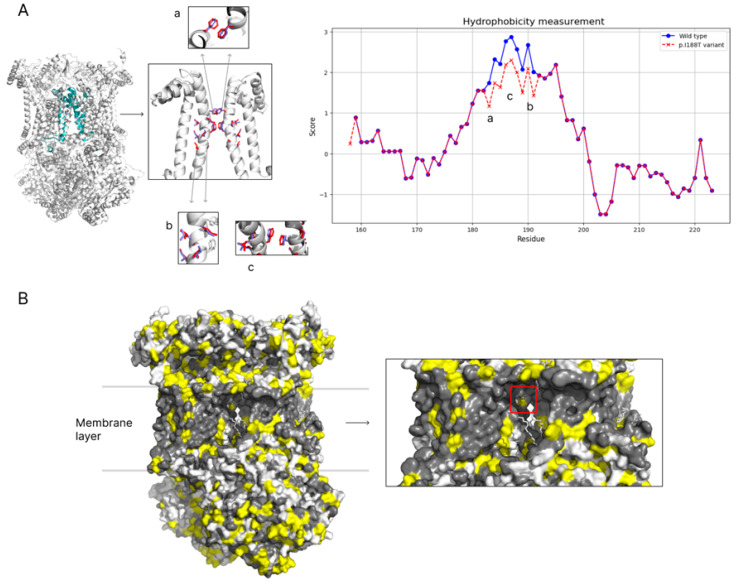
(**A**) Hydrophobicity analysis highlights a significant reduction in hydrophobicity at residues 188–191 in the mutated protein compared to the wild type. (**B**) The molecular surface representation reveals that the mutation alters the local chemical environment of the transmembrane region. The mutated residues are highlighted, showing a disruption in the uniform hydrophobic surface, with polar patches emerging due to the threonine substitution.

The observed variant *MT-CYB*:m.15309T>C results in an amino acid substitution at position 188 in the mitochondrial cytochrome B. The variant I188T has been reported once in the ClinVar database to have uncertain significance (accession number SCV000998316, accessed on 22 November 2024) according to criteria for reporting clinical significance of mitochondrial variants in line with the Modified ACMG Guidelines for mt-tRNA-1. Algorithmic in silico prediction of variant significance provides conflicting conclusions. PolyPhen and SIFT predict I188T as probably damaging and deleterious with low confidence, respectively. On the other hand, AlphaMissense reports the variant as benign. Similarly, variant significance estimation based on protein region conservation between species shows I188T to be likely benign. To investigate the structural and functional impact of the p.I188T variant in the mitochondrial cytochrome b (MT-CYB) protein, computational modeling was performed using AlphaFold2 to generate structures of both the wild type and protein with the p.I188T variant. A substitution of the hydrophobic isoleucine with the polar threonine at position 188 occurs within the transmembrane region of MT-CYB (Figure 10A). Kyte–Doolittle hydrophobicity analysis revealed a significant decrease in hydrophobicity at residues 188–191 in the mutated protein compared to the wild type (Figure 10A), suggesting destabilization of protein–lipid interactions crucial for membrane anchoring and structural integrity. Surface visualization further demonstrated the emergence of polar patches in the transmembrane region, disrupting the uniform hydrophobic surface and potentially interfering with protein folding and complex assembly (Figure 10B). Given that complex III functions as a dimer, the presence of a p.I188T variant at two sites within complex III amplifies its destabilizing effect. Moreover, positions 178 to 198 in the MT-CYB protein are key transmembrane regions. Thus, I188T may also affect the protein’s ability to properly localize into the mitochondrial membrane. Finally, this transmembrane region contains two significant histidine amino acids at positions 182 and 196. These histidine amino acids are crucial in binding heme B, facilitating MT-CYB function. I188T is located between these two key positions and any loss in structural stability or failure to localize in the membrane has the potential to majorly affect the protein’s function and thus lead to pathogenetic changes consistent with the observed phenotype.

## 4. Discussion

We presented a detailed phenotypic analysis of a Slovenian family with three individuals, of whom one suffered from LHON-like optic atrophy and two were unaffected. The presented affected individual had a profound visual loss at an early age (19 years) with three visual acuity drops and improvements in the left eye before the final visual acuity loss over the course of eight months. The visual loss was characterized by centrocecal scotoma, abnormal PERG N95 and VEP and retinal nerve fiber thinning observed via OCT. After two years of legal blindness, the patient experienced visual acuity improvement, which reached RE 0.5 and LE 0.7 Snellen (0.3 and 0.15 LogMAR) over the course of five years. There was also improvement in microperimetry, visual fields and electrophysiology characteristics. Our patient had a presentation atypical for LHON with a series of spontaneous visual acuity improvements, whilst exacerbations were observed after intravenous corticosteroid treatments. Atypical features for LHON were also late development of pallor of the optic disk and relatively late onset of color vision deficiency. Spontaneous recovery was also reported over time in the patient with LHON. This was associated with larger optic disks, childhood-onset LHON, mutation 14,484 and arLHON [8,15,16]. However, the initial fluctuation of relatively rapid improvements and exacerbations was a distinct feature in our patient and made clinical management especially difficult.

Mitochondrial sequencing showed the presence of a *MT-CYB*:m.15309T>C variant in proband’s mtDNA with a high heteroplasmy level (65%), which introduced an Ile188Thr amino acid change in the invariant position of the highly conserved domain of the MT-CYB, affecting complex III. Segregation confirmed the same variant in the mother and two sisters of the patient, who have not yet been affected.

The *MT-CYB*:m.15309T>C missense variant has been reported once in ClinVar database as a variant of unknown significance and five times in the HelixMTdb. In a Slovenian database of 16,000 exomes + genomes, this variant was found in three individuals with other referral diagnoses, in addition to our four family members. In one with retinal dystrophy and night blindness, where the causal variant was RHO Gly90Asp, the gene was 100% homoplasmic. In the patient’s healthy mother, it was present with 28% heteroplasmy. It was also found in a patient with intestinal perforation (with 57% heteroplasmy), who had a likely pathogenic causal variant in COL3A1. Although this variant is present in three unrelated subjects with different referral reasons in our database and is reported five times in HelixMTdb (once in homoplasmy and four times in heteroplasmy), we believe that this frequency does not preclude its pathogenicity, as, for example, the typical pathogenic variant m.11778A>G is even more frequent in HelixMTdb (44 times in homoplasmy and 34 times in heteroplasmy).

Mitochondrial respiratory function has been shown to be decreased in the skin fibroblasts of LHON patients compared to healthy controls [17,18,19,20,21,22], with some exceptions [23]. The mutations causing LHON in these studies are heterogeneous, with some studies using patients with the most common, previously reported mutations and others aiming to confirm the status of newly found mutations as pathogenic with this functional analysis. Studies on cybrid cell lines show that LHON-causing mutations result in decreased mitochondrial respiration or complex I function [22,24,25,26,27]. Mitochondrial function of PBMCs was reported to be decreased in LHON [28] and LHON-like optic neuropathy [29] based on resazurin fluorescence or decreased ATP synthesis and complex I function in lymphoblast cell lines [30]. Studies reporting direct measurement of mitochondrial respiration in blood cells of LHON patients in the current literature are few and inconsistent [25,31]. It is known that respiratory complex III is required for complex I stability in mammalian mitochondria and that mutations in the mtDNA-encoded cytochrome *b* gene are associated either with combined complex I+III deficiency or with only complex III deficiency [32]. Our study shows that the respiration of intact cells and complex I-linked respiration in the PBMCs of the patient were lower compared to his age- and sex-matched control. We also confirmed increased production of superoxide anions and mitochondrial membrane depolarization in the PBMCs of the patient with LHON compared to the healthy control’s PBMCs, which is in line with previously reported findings [33,34]. These findings imply that this novel variant compromises mitochondrial respiration, as well as mitochondrial membrane potential and ROS production, even though the lack of complex III activity measurement presents a limitation of this study.

The mitochondrial cytochrome b (MT-CYB) protein is a key component of complex III in the electron transport chain. A substitution of the hydrophobic isoleucine with the polar threonine at position 188, occurs within the transmembrane region of MT-CYB, a critical hydrophobic environment necessary for maintaining protein stability and proper function. Kyte–Doolittle hydrophobicity analysis revealed a significant decrease in hydrophobicity at residues 188–191 in the mutated protein compared to the wild type (Figure 10A), suggesting destabilization of protein–lipid interactions crucial for membrane anchoring and structural integrity within the protein core. The mutation introduces a hydrophilic residue (threonine) into a hydrophobic environment, potentially destabilizing the protein–lipid interactions essential for membrane anchoring and protein folding.

Surface visualization of mutated complex III further demonstrated the emergence of polar patches in the transmembrane region, disrupting the uniform hydrophobic surface and potentially interfering with protein folding and complex assembly (Figure 10B). Given that complex III functions as a dimer, the presence of a p.I188T variant at two sites within complex III amplifies its destabilizing effect, likely compromising the overall stability and activity of the electron transport chain. An impaired electron transfer process can lead to reduced efficiency and increased production of reactive oxygen species (ROS), as we proved in our experiments. Amplification of the mutation impact due to its presence at two sites in the protein complex may further exacerbate functional impairments, possibly contributing to mitochondrial dysfunction, and might be associated with the imbalance between dysfunctional and normal protein production, which may be reflected in the patient’s fluctuations in vision in the early phases of the disease. The observed adverse effect of corticosteroids in this respect is worth a note as it has been reported that a high dosage of corticosteroids may negatively affect mitochondrial function [35].

SIFT2 predicts the mutation as likely pathogenic with a score of 0.01 (lower scores indicate greater severity), while PolyPhen2 classifies it as very likely harmful with a score of 0.954 (higher scores indicate greater severity). Conversely, a newly developed deep learning algorithm for predicting the pathogenicity of missense mutations, AlphaMissense, predicted the p.I188T variant to be benign with a score of 0.1776. It is important to note that AlphaMissense based its prediction solely on the isolated MT-CYB domain. However, the p.I188T variant exists in the context of complex III as part of an MT-CYB dimer, amplifying the mutation’s effects due to its presence in two sites within the dimeric structure. Therefore, it is crucial to adopt a broader approach that considers the mutation’s impact within the full protein complex. The integration of bioinformatic tools and structural analyses provides mechanistic insights into the pathogenicity of the p.I188T variant, highlighting its role in disrupting the structural integrity of MT-CYB and contributing to mitochondrial dysfunction.

## 5. Conclusions

Herein we present a novel variant of unknown significance in CYT-B of a Slovenian family (son (patient), mother and daughters) with one affected individual with an LHON-like phenotype and bilateral optic neuropathy. Based on the phenotype of the patient, segregation analysis, mitochondrial function assessment and proteomic analysis, we propose that this variant might be causative in the presented case.

## Data Availability

The original contributions presented in this study are included in the article and Supplementary Materials. Further inquiries can be directed to the corresponding author.

## Supplementary Materials

The following supporting information can be downloaded at https://www.mdpi.com/article/10.3390/genes16010108/s1. Figure S1. Head MRI showing hyperintense signal along the optic nerves R>L and small effusion within the ON sheath consistent with perineuritis of both optic nerves R>L. There are no signs of demyelination; Figure S2. Microperimetry (MP1, fast strategy) at the disease onset and after improvement; more stimuli were detected; Figure S3. Optical coherence tomography showed no structural improvement in the thickness of RNFL and GCL, not even in the places where there was an improvement in microperimetry in the right eye; Figure S4. Optical coherence tomography showed no structural improvement in the thickness of RNFL and GCL, not even in the places where there was an improvement in microperimetry in the left eye; Figure S5. Peripapillary RNFL showed thinning in all quadrants with relative preservation of the inferior nasal quadrant; Figure S6. VEP showed no improvement in VEP P100 amplitude with visual acuity improvement; Figure S7. Pattern ERG showed improvement in the N95 amplitude. Figure S8. Pattern ERG showed improvement in the P50 amplitude, also probably due to better fixation; Figure S9. Family pedigree with variant heteroplasmy percentage.
