# Supplementary figures and images for: Atypical Leber Hereditary Optic Neuropathy (LHON) Associated with a Novel MT-CYB:m.15309T>C(Ile188Thr) Variant
Source: Genes (Basel). 2025 Jan 20;16(1):108. doi: 10.3390/genes16010108 (PMC11764998; doi:10.3390/genes16010108)

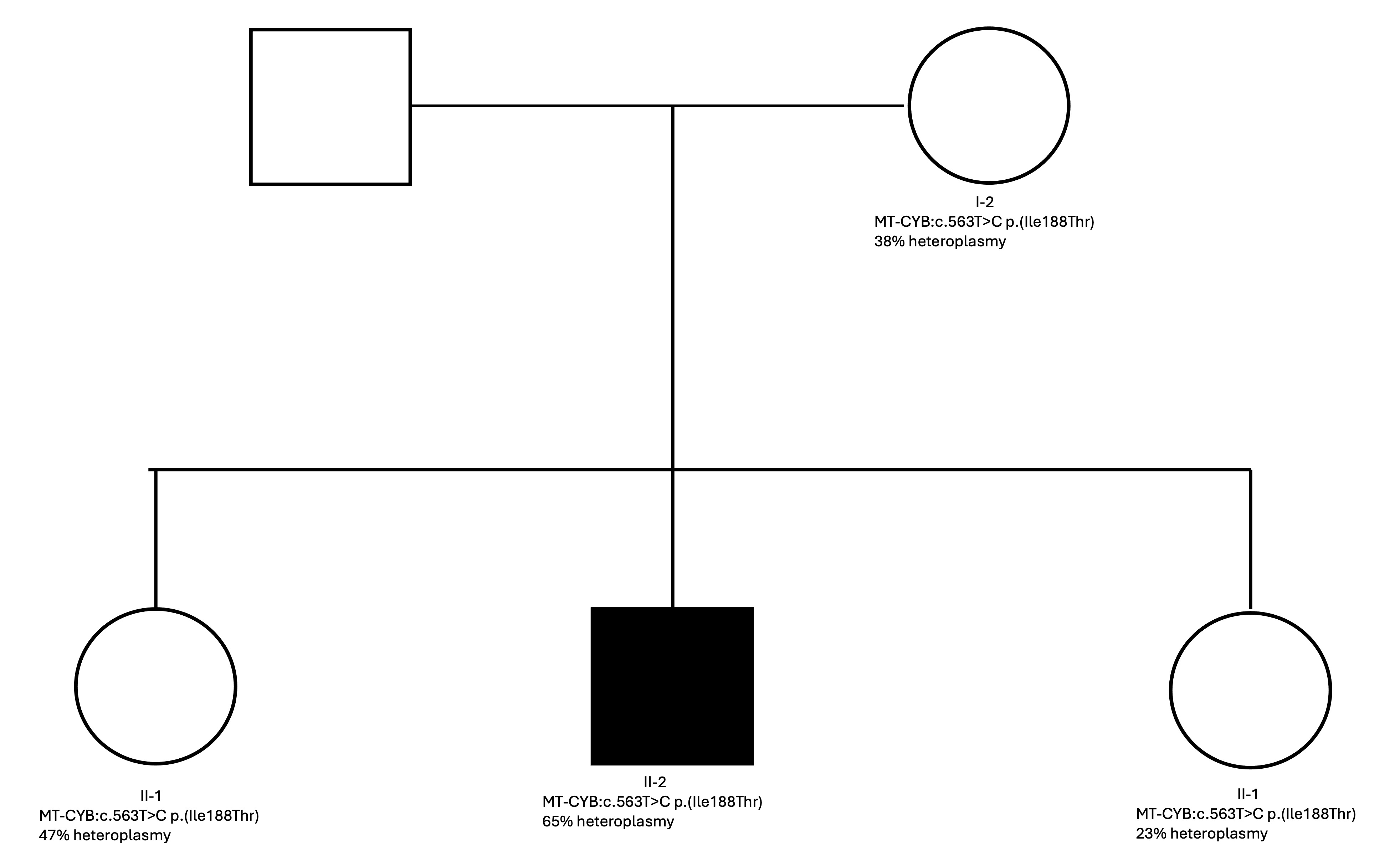

Supplement: Supplementary file 1 [file genes-16-00108-s001.zip › Supplementary Genes Revision/Supplementary figure S9.jpg]

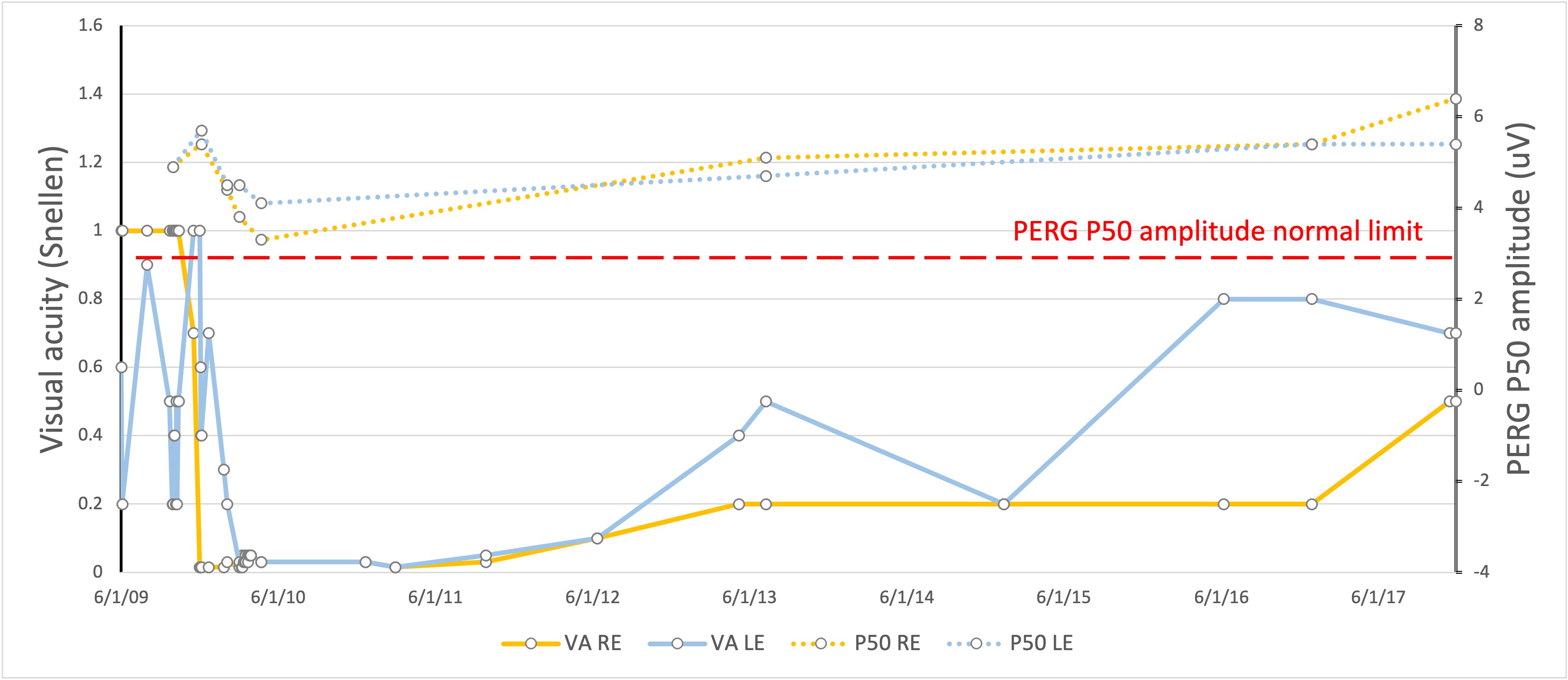

Supplement: Supplementary file 1 [file genes-16-00108-s001.zip › Supplementary Genes Revision/Supplementary figure S8.jpg]

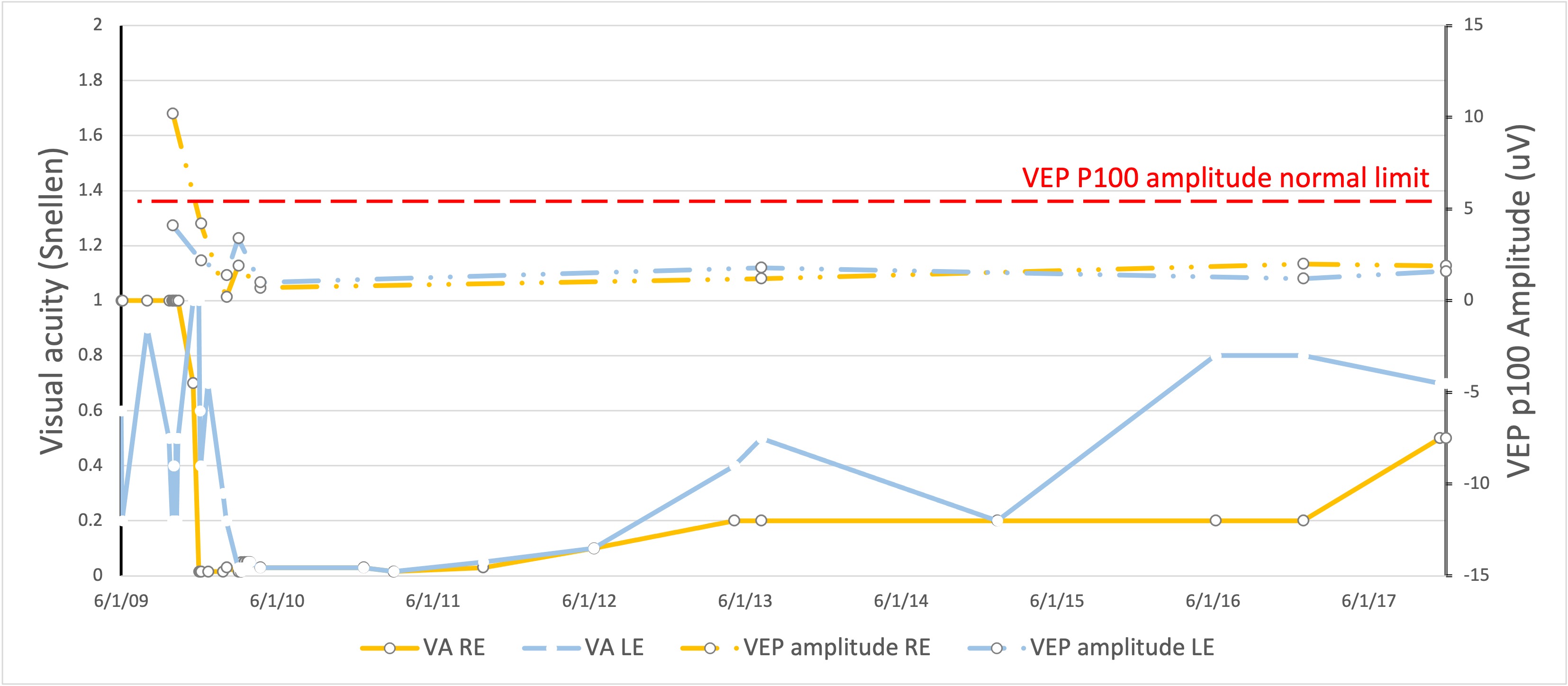

Supplement: Supplementary file 1 [file genes-16-00108-s001.zip › Supplementary Genes Revision/Supplementary figure S6.jpg]

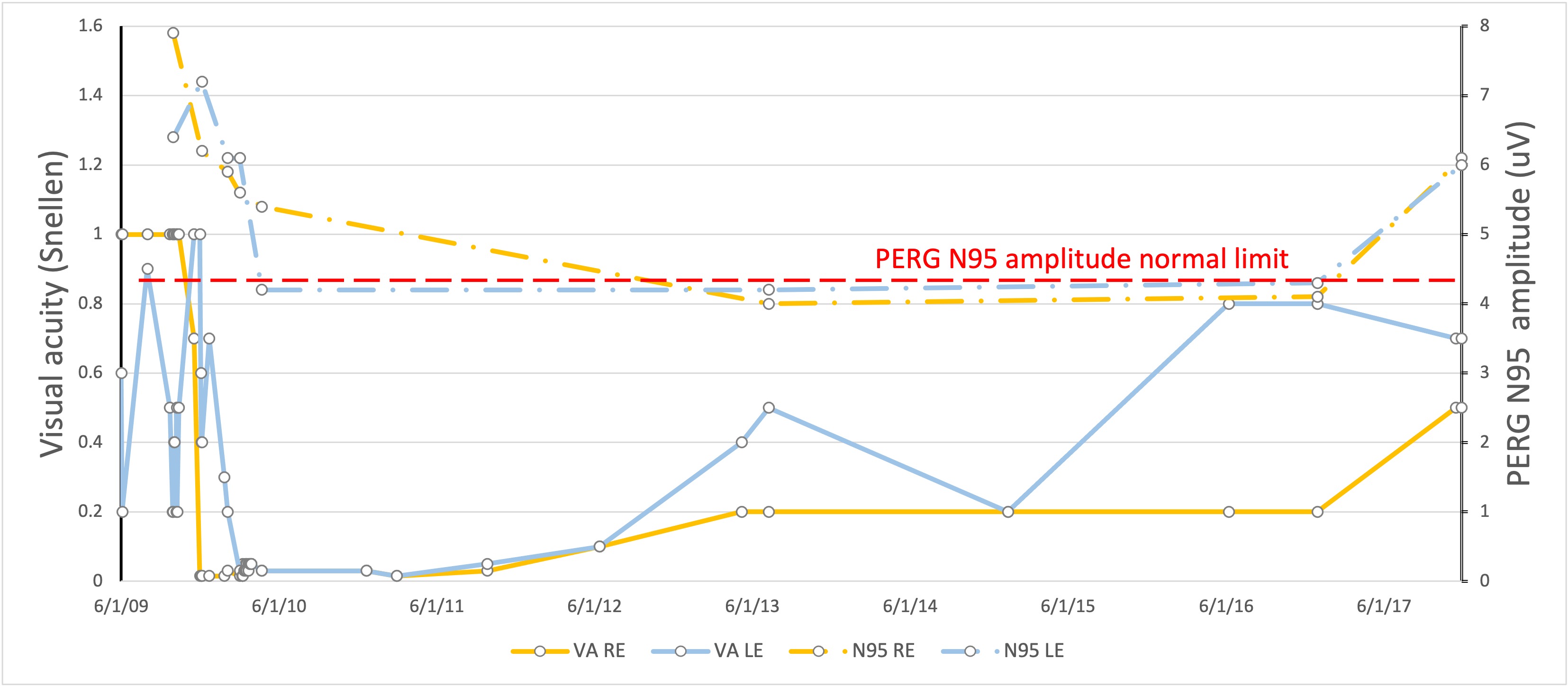

Supplement: Supplementary file 1 [file genes-16-00108-s001.zip › Supplementary Genes Revision/Supplementary figure S7.jpg]

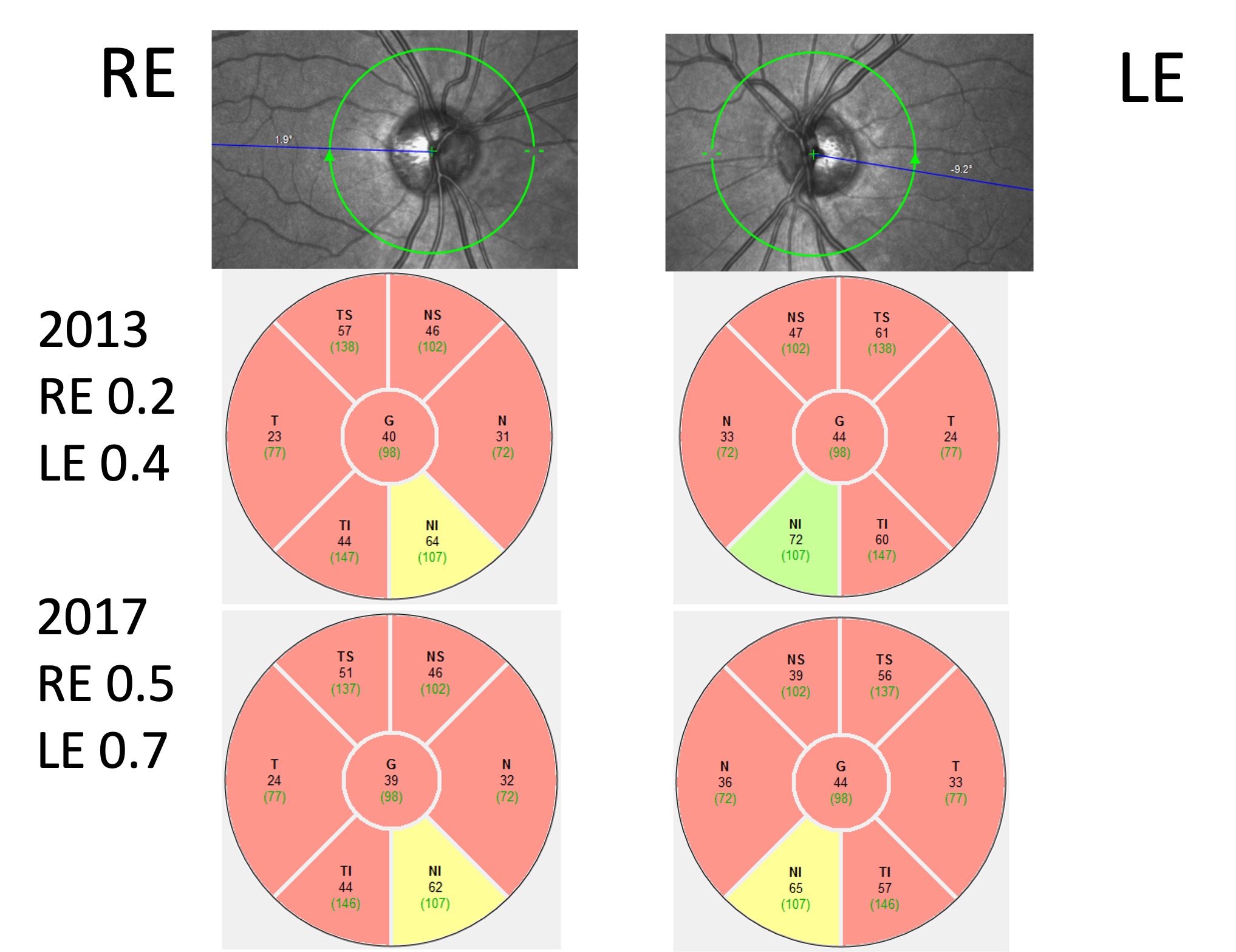

Supplement: Supplementary file 1 [file genes-16-00108-s001.zip › Supplementary Genes Revision/Supplementary figure S5.jpg]

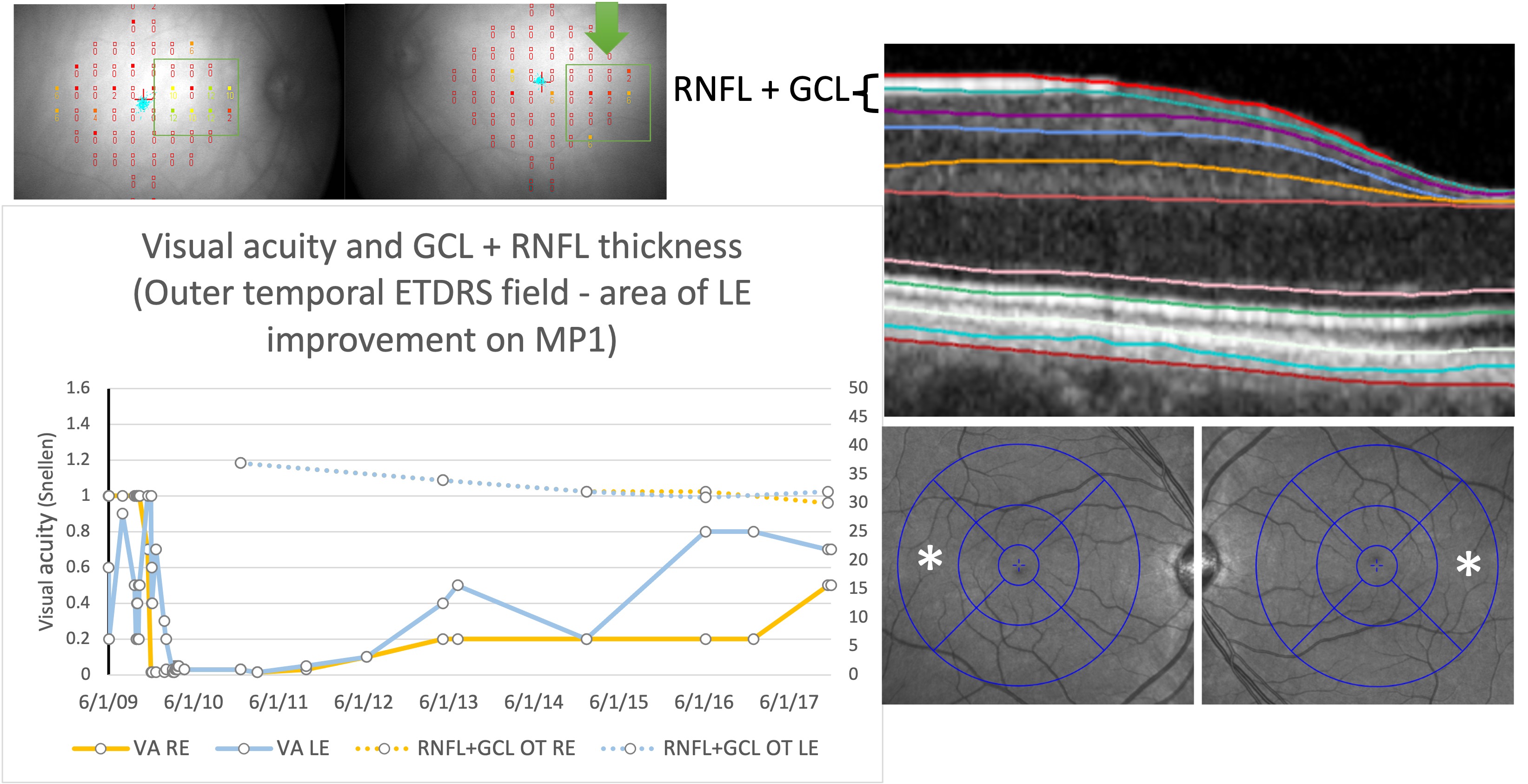

Supplement: Supplementary file 1 [file genes-16-00108-s001.zip › Supplementary Genes Revision/Supplemetary figure S4.jpg]

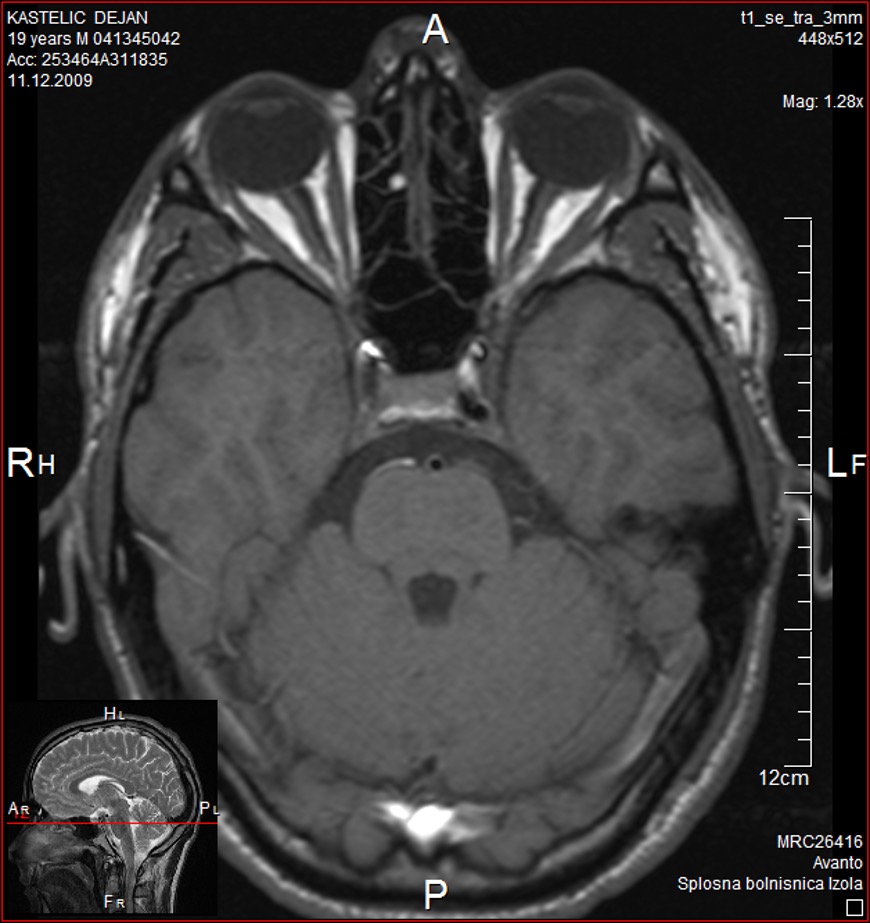

Supplement: Supplementary file 1 [file genes-16-00108-s001.zip › Supplementary Genes Revision/Supplementary Figure S1.jpg]

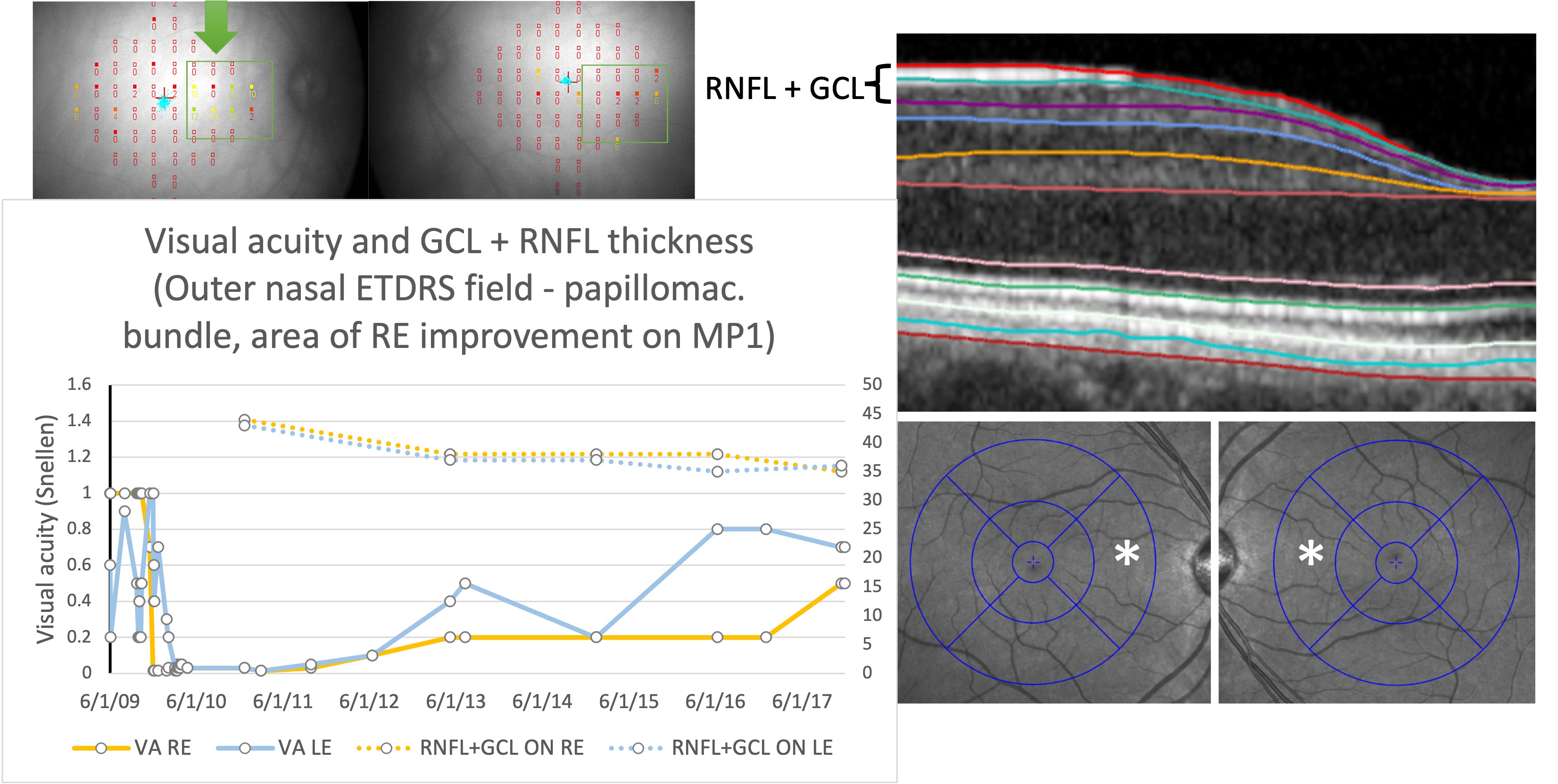

Supplement: Supplementary file 1 [file genes-16-00108-s001.zip › Supplementary Genes Revision/Supplementary figure S3.jpg]

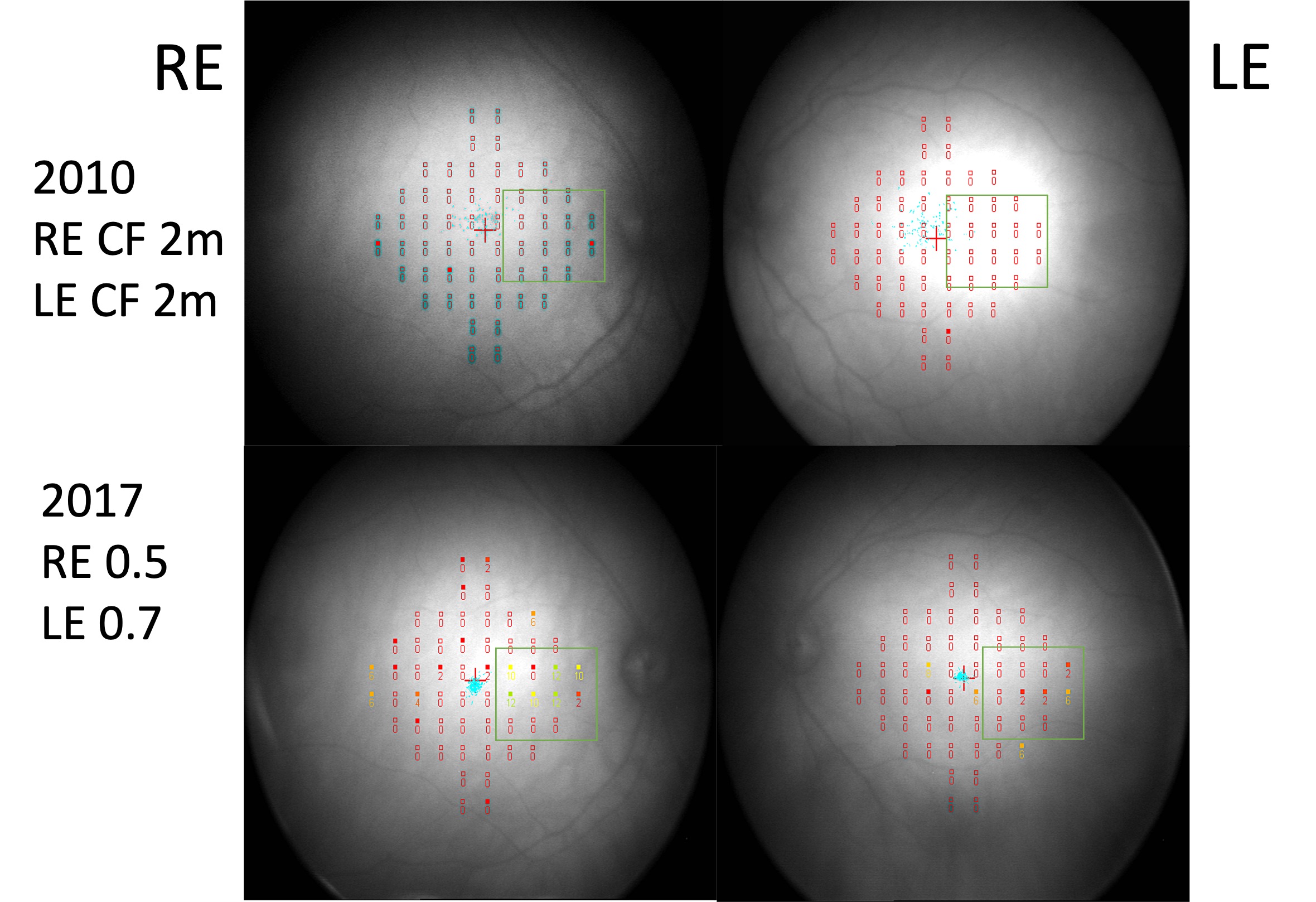

Supplement: Supplementary file 1 [file genes-16-00108-s001.zip › Supplementary Genes Revision/Supplementary figure S2.jpg]
